# Assessment of 6MV flattening filter free (FFF) beam commissioning parameters for Versa HD and TrueBeam: A comparison study

**DOI:** 10.1002/acm2.70723

**Published:** 2026-07-29

**Authors:** Mustafa K. Al‐Aseebee, Hussien Mraity, Marco Esposito

**Affiliations:** ^1^ Department of Physics Faculty of Science University of Kufa Al‐Najaf Iraq; ^2^ Middle Euphrates Cancer Center Al‐Najaf Iraq; ^3^ Amal Al‐Hayat Hospital Al‐Najaf Iraq; ^4^ S.O.C. Fisica Sanitaria Firenze‐Empoli Azienda USL Toscana Centro Bagno a Ripoli Italy; ^5^ Medical Physics The Abdus Salam International Center for Theoretical Physics Trieste Italy

**Keywords:** beam profile, flattening filter free (FFF), output factor (OF), percentage depth dose (PDD), treatment planning study, TrueBeam, unflatness conversion index (UCI), Versa HD

## Abstract

**Background:**

Flattening filter‐free (FFF) beams are increasingly deployed for stereotactic body radiotherapy (SBRT) and stereotactic radiosurgery (SRS). The Elekta Versa HD (VHD) and Varian TrueBeam (TB) are the two most widely used FFF‐capable platforms; however, no prior study has compared their 6 MV FFF commissioning parameters under fully equipment‐equivalent conditions.

**Purpose:**

To quantify the dosimetric differences between the Elekta VHD and Varian TB 6 MV FFF platforms under equipment‐equivalent conditions and to assess their translation into patient treatment plan parameters.

**Methods:**

Percentage depth doses (PDDs), lateral beam profiles, and output factors (OFs) were acquired using an identical‐model IBA SMARTSCAN three‐dimensional water tank, a unified detector set (IBA RAZOR for small fields; CC13 for standard fields), and a fixed source‐to‐surface distance of 90 cm for both platforms, in conformance with AAPM TG‐45, AAPM TG‐106, and IAEA TRS‐483. A treatment planning translation study was performed in the Monaco TPS using the IBA IMRT phantom, with 3D‐CRT plans generated for four spherical targets (1, 3, 6, and 12 cm diameter) on both beam models.

**Results:**

VHD demonstrated consistently deeper beam penetration, with R50 exceeding TB by a mean of 1.753 ± 0.048 cm across all field sizes (*t*(9) = 36.34, *p* < 0.001, Cohen's *d* = 11.49, 95% CI [1.643, 1.862] cm). TB delivered significantly higher surface doses than VHD (mean Ds: 63.64 ± 5.77% vs. 48.78 ± 7.72%; *p* = 0.002, |*r*| = 1.000). At 10 cm depth, VHD showed a higher percentage depth dose (mean difference = 5.005 ± 0.352%; *t*(9) = 14.22, *p* < 0.001, *d* = 4.498). The Unflatness conversion index (UCI) was strongly correlated with field size (*r* = 0.916, *p* = 0.001) and statistically independent of measurement depth (*p* = 0.292). At the plan level, TB required 5.9–17.6% more monitor units (MU) than VHD across all target sizes, with the differential diminishing as target diameter increased. Surface dose differences were preserved across all four targets (TB − VHD: 15.9–31.1%). Both the homogeneity index and gradient index reversed direction between the 1 cm target (VHD superior) and targets of 3 cm and larger (TB superior), consistent with the field‐size dependence of UCI.

**Conclusion:**

A harmonized, equipment‐equivalent measurement approach demonstrated systematic differences between VHD and TB FFF beams in penetration, surface dose, and field‐size‐dependent unflatness. Treatment planning study across four target sizes (1–12 cm) showed these differences propagate to plan‐level parameters in a target‐size‐dependent manner, with VHD advantageous for small targets and TB for larger ones. The UCI, depth‐independent and strongly correlated with field size (*r* = 0.916), provides a simple cross‐platform unflatness comparison tool supporting machine‐specific planning strategies in multi‐platform clinical settings.

## 1 INTRODUCTION

Advances in radiation therapy have contributed to improved cancer survival rates and reduced treatment‐related morbidity.^1^ Flattening‐filter‐free (FFF) photon beams have attracted increasing clinical interest, particularly for stereotactic body radiotherapy (SBRT) and stereotactic radiosurgery (SRS).^2^, ^3^ The removal of the flattening filter enables higher dose rates and shorter treatment times, reducing intra‐fraction motion and improving treatment efficiency.^4^ At the same time, FFF beams exhibit a characteristic nonuniform dose profile, lower peripheral dose, and increased surface dose, all of which must be accurately characterized for safe clinical implementation.^5^, ^6^, ^7^

The Elekta Versa HD (VHD) and Varian TrueBeam (TB) systems are among the most widely used platforms capable of delivering 6 MV FFF photon beams. Although both systems operate at the same nominal energy, their beam‐generation architectures differ in a clinically relevant manner. In TB, a thin brass plate replaces the flattening filter while maintaining an electron beam configuration similar to that of flattened beams. In VHD, a thicker stainless‐steel disc is used in combination with different radio‐frequency and electron gun settings, resulting in distinct energy spectra between beam modes.^8^ These design differences are associated with measurable variations in beam penetration, surface dose, and off‐axis profile characteristics, which must be accurately represented during commissioning and beam modelling.

Previous studies have investigated FFF beam characteristics across different platforms. Fogliata et al. established key parameters for FFF beam quality assurance, including unflatness and slope, and demonstrated inter‐platform differences in beam shape and depth‐dose characteristics.^9^, ^10^ Palanivelu et al. extended these comparisons across multiple vendors, reporting differences in surface dose, penumbra, and collimation effects.^11^ Hao et al. explored alternative measurement approaches for beam study, highlighting correlations between profile shape and depth‐dose behavior across platforms,^12^ while multicenter studies such as Casar et al. have investigated detector‐dependent variability in small‐field dosimetry.^13^

Despite this body of work, standardized comparisons between VHD and TB under identical measurement conditions remain limited. Many published studies involve different detectors, water tank systems, or acquisition geometries across institutions, making it difficult to isolate true platform‐dependent differences from measurement‐related variability.^11^, ^12^, ^13^ As a result, although general trends are recognized, direct attribution of observed differences to beam generation and delivery characteristics is often uncertain.

In addition to this methodological limitation, an important clinical aspect remains insufficiently addressed. In many radiotherapy departments, multiple linear accelerators from different vendors operate within the same clinical workflow. In these settings, understanding and quantifying dosimetric differences between platforms is not only relevant for commissioning but also for ensuring consistency in treatment planning and for optimizing patient allocation across machines. However, most existing studies focus on physical beam characterization, with limited investigation of how these differences propagate into treatment planning or influence clinically relevant plan parameters.

The present study was designed to address both aspects. Commissioning parameters of 6 MV FFF beams from VHD and TB were measured using identical equipment and acquisition protocols to minimize measurement‐related variability. In addition, a treatment planning study was performed to assess how these differences are reflected at the plan level. The study also introduces the Unflatness Conversion Index (UCI) as a simple descriptor for quantifying inter‐platform differences in beam profile characteristics. The overall aim is to provide a practical framework for evaluating dosimetric differences between linear accelerators within the same clinical environment and to assess their potential implications for treatment planning and delivery.

## 2 MATERIALS AND METHODS

### 2.1 Equipment and study design

Measurements were acquired from the 6 MV FFF beams of a Varian TB and an Elekta VHD linear accelerator are summarized in Table 1. All beam scanning and data collection were performed using the IBA SMARTSCAN three‐dimensional water tank (IBA, Louvain‐la‐Neuve, Belgium; scanning volume 48 × 48 × 41 cm^3^).^14^ The use of the same type of water tank system for both platforms eliminated inter‐equipment measurement bias, reducing measurement‐related variability and minimizing measurement‐system dependence in the comparison. All measurements followed the Elekta manufacturer's reference manual and AAPM Task Group (TG) Reports No. 45 and No. 106.^15^, ^16^

**TABLE 1:** Characteristics of VHD and TB.

Characteristic	VHD (Agility™)	TB (Millennium 120)
FF substitute (6 MV FFF)	2 mm stainless‐steel disc	0.8 mm brass plate
Beam generation	Separate RF/gun settings for FFF and flattened beams	Shared electron beam for both FFF and flattened beams
MLC design (leaves/pairs)	160 leaves (80 pairs) Uniform 5 mm width across full 40 × 40 cm^2^ aperture	120 leaves (60 pairs), 80 central leaves at 5 mm; 20 outer leaves at 10 mm per bank Max field: 40 × 40 cm^2^
MLC spatial resolution	Uniform across full aperture	Higher resolution centrally; coarser peripherally
Jaw / MLC arrangement	MLC replaces the jaws entirely (no backup jaws or diaphragms)	Jaws positioned upstream of MLC (tertiary collimator)

All measurements on both platforms were performed by the same operator using identical acquisition protocols, software settings, and detector configurations, eliminating inter‐operator variability as a confounding factor. Temperature and pressure corrections were applied identically to both platforms.

### 2.2 Beam data collection: PDDs and profiles

PDDs and lateral beam profiles were measured in accordance with the Elekta Monaco treatment planning system (TPS) reference manual for beam modeling. All measurements were conducted at a fixed SSD of 90 c, as specified by the Elekta Monaco TPS beam modeling reference manual for VHD commissioning. FSs ranged from 2 × 2 cm^2^ to 40 × 40 cm^2^. The effective point of measurement was automatically adjusted using myQA Accept software,^17^ and the central axis of the water phantom was verified before each measurement session. Measurement reproducibility was verified by repeated acquisitions at two reference field sizes (5 × 5 and 10 × 10 cm^2^) until consistent readings were obtained in the same direction, confirming measurement stability prior to data collection. The following parameters were derived from each PDD curve: depth of maximum dose (Dmax), depth at 50% dose (R50), depth at 80% dose (R80), surface dose (Ds, at 0.0 mm depth), and percentage depth dose at 5 cm (D5), 10 cm (D10), and 20 cm (D20) depth.

The choice of detector followed the FS: for FSs ≤ 3 × 3 cm^2^, an IBA RAZOR chamber (field detector) and an IBA Stealth chamber (reference detector) were employed; for FSs ≥ 4 × 4 cm^2^, an IBA CC13 chamber served as both field and reference detector. The RAZOR chamber was selected for small‐field measurements owing to its high spatial resolution and reduced sensitivity to the partial volume effect.^18^ Detector characteristics are summarized in Table 2.

**TABLE 2:** Detectors used for beam data measurements and their corresponding dosimetric characteristics.

Detector	Role	Active Vol. (cm^3^)	Cavity radius (mm)	Wall material	Wall thickness (g/cm^2^)	Water approved
IBA RAZOR Chamber	Field detector	0.01	1.0	C‐552	0.088	Yes
IBA Stealth Chamber	Reference detector	—	—	C‐552	—	No
IBA CC13	Field + reference	0.13	3.0	C‐552	0.07	Yes

Crossline (CL) and inline (IL)profiles were measured at depths of 5, 10, and 20 cm.^17^ For FSs exceeding the water tank scanning dimension (40 × 40 cm^2^), one half‐profile was acquired and the contralateral half was mirrored using the acquisition software. All PDDs and profiles were smoothed with a median filter using a 0.5 cm sliding window.^19^

Paired gamma analyses were performed using criteria of 3%/1 mm global with the monaco commissioning utility (MCU) software to quantify inter‐platform agreement. The 3%/1 mm criterion was deliberately selected over the more permissive 3%/2 mm because this study included small FS with steep dose gradients; the tighter 1 mm distance‐to‐agreement tolerance reveals subtle inter‐platform profile and penumbral differences that a 2 mm tolerance would otherwise mask.^20^ Four profile metrics were calculated for each FS and depth:

**(i) Unflatness** — the ratio of the central‐axis dose to the dose at a predefined off‐axis distance (80% of the FS half‐width), as defined by Fogliata et al.^9^ (Equation 1):
(1) $$\mathrm{Unflatness}=\frac{Dose_{central.axis}}{Dose_{X.off-axis}}$$
**(ii) UCI** — a novel cross‐platform index introduced in the present study to quantify the systematic unflatness divergence between VHD and TB (Equation 2):
(2) $$\mathrm{UCI}=\frac{\mathrm{Unflatnes}s_{\left(Versa\;HD,FS,d\right)}}{\mathrm{Unflatnes}s_{\left(TrueBeam,FS,d\right)}}$$

A UCI of 1.000 indicates equivalent beam unflatness between platforms. Values exceeding 1.000 indicate greater unflatness in VHD; values below 1.000 indicate greater unflatness in TB. UCI was calculated separately for the CL (UCI_CL) and IL (UCI_IL) directions and as a mean value (UCI_Mean).

**(iii) Slope** — the gradient of a line connecting fixed points at one‐third and two‐thirds of the half‐beam width, following Fogliata et al.^9^ (Equation 3):
(3) $$\mathrm{Slope}=\frac{\left(x_{1}-x_{2}\right)\times \left(y_{1}-y_{2}\right)}{\left(x_{1}-x_{2}\right)2},$$

where (*x*1, *y*1) and (*x*2, *y*2) are dose‐position coordinates at −2/3 and −1/3 of the left half‐profile (or +1/3 and +2/3 of the right half‐profile), respectively.

**(iv) Penumbra width** — defined as the lateral distance between the 80% and 20% isodose levels, consistent with AAPM TG‐106.^16^ Profile normalization was performed with respect to the inflection point (assigned a reference value of 50%) to improve penumbra stability, particularly in small‐field and MLC‐defined apertures.^9^, ^20^

### 2.3 Output factor measurements

OFs were measured at a depth of 10 cm and SSD of 90 cm. For FSs from 1 × 1 to 5 × 5 cm^2^, the IBA RAZOR detector was used in accordance with the IAEA TRS‐483 small‐field dosimetry protocol^21^; for FSs from 7 × 7 to 40 × 40 cm^2^, the CC13 ionization chamber was used. All readings were normalized to the 10 × 10 cm^2^ reference field.^17^ Output correction factors applied to the RAZOR chamber ranged from *k* = 1.004 at 1 × 1 cm^2^ to *k* = 1.000 at 5 × 5 cm^2^.^22^

### 2.4 Statistical analysis

Prior to parametric testing, normality of inter‐platform differences was assessed using the Shapiro–Wilk test for each dosimetric parameter. For normally distributed parameters, paired *t*‐tests were applied to compare VHD and TB measurements across the ten FS (*n* = 10 pairs). For surface dose (Ds), where normality was violated, the Wilcoxon signed‐rank test was used as a nonparametric alternative; the Hodges–Lehmann estimator was reported as the measure of central tendency. Effect sizes were quantified using Cohen's *d* for parametric tests and the matched rank biserial correlation *r* for the Wilcoxon test. For UCI analysis, Pearson correlation quantified the relationship between UCI_Mean and FS (*n* = 8 FS), and repeated measures ANOVA with Greenhouse‐Geisser correction examined the effect of measurement depth (5, 10, and 20 cm) on UCI; Mauchly's test was used to assess the sphericity assumption. All analyses were performed using JASP (version 0.16). Statistical significance was set at α = 0.05. For FS were treated as the sampling unit for paired comparisons. Readers should note that measurements across FS are not fully independent, as beam characteristics vary systematically with aperture.

### 2.5 Treatment planning study

**FIGURE 1:**
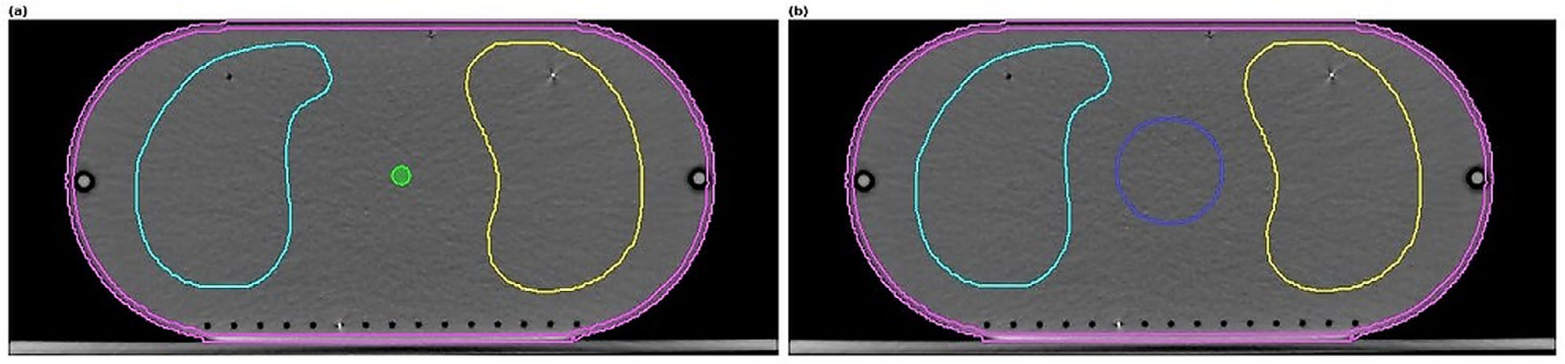
Axial CT slice of the IBA IMRT phantom showing the delineated planning target volumes used for the treatment planning study: (a) 1 cm PTV (small target) and (b) 6 cm PTV (medium‐sized target).

To examine whether the dosimetric differences identified during commissioning carry through to treatment plan differences, a treatment planning study was performed in the Monaco TPS using the IBA IMRT phantom. Fours spherical PTVs, of 1, 3, 6 and 12 cm in diameter, were delineated to represent small‐field (SRS/SBRT) and conventional target geometries (Figure 1).

A 3D conformal plan was generated for each PTV on both the VHD and TB beam models, using a matched beam arrangement of eight equally spaced fields at 45° gantry angle intervals (0°, 45°, 90°, 135°, 180°, 225°, 270°, 315°), equal field weighting, and prescription normalization to D95%, with the beam model as the sole variable between paired plans. Surface dose was assessed at a depth of 3 mm beneath the phantom surface. Plan quality was assessed using D95%, D2%, the homogeneity index, defined per ICRU Report 83 as HI = (D2% − D98%)/D50% 33, the conformity index (CI = V100%/V_PTV), and the percentage surface dose.

## 3 RESULTS

### 3.1 Percentage depth dose curves

**FIGURE 2:**
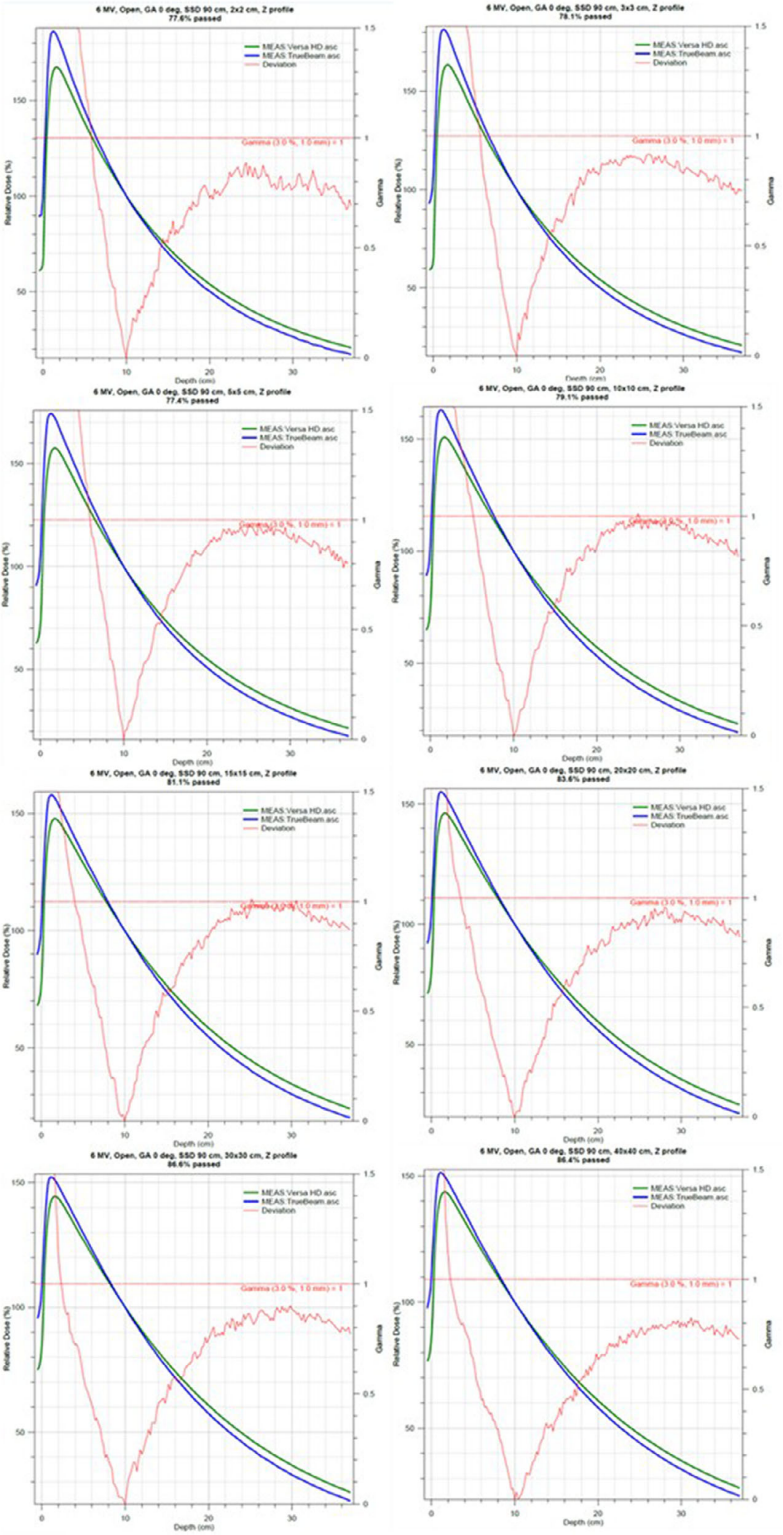
A comparison between the PDDs for TB and VHD for different FSs.

Figure 2 presents the PDD comparison curves and corresponding gamma analysis results. For small FSs (2 × 2–5 × 5 cm^2^), gamma pass rates were 78%. For medium FSs (10 × 10 to 20 × 20 cm^2^), pass rates improved to 79%–84%, reflecting greater depth‐dose agreement and reduced measurement noise at larger apertures. The 30 × 30 cm^2^ FS produced the highest pass rate of 86.6%, while the 40 × 40 cm^2^ FS showed a marginal decline, attributable to increased lateral scatter and MLC transmission at large apertures.

Paired *t*‐test analysis demonstrated that VHD exhibited significantly greater beam penetration than TB across all FS (R50: mean VHD = 14.80 ± 1.41 cm versus mean TB = 13.05 ± 1.54 cm; mean difference = 1.753 ± 0.048 cm; *t*(9) = 36.34, *p* < 0.001, Cohen's *d* = 11.49, 95% CI [1.643, 1.862 cm]). The Shapiro–Wilk test confirmed that R50 differences were normally distributed (*W* = 0.899, *p* = 0.216). This systematic penetration advantage of 1.45–1.92 cm across all FSs (Table 3) is consistent with the distinct radio frequency (RF) and electron gun parameters employed by VHD in FFF mode, which produce a harder effective photon spectrum than the brass‐plate filter approach used by TB.

**TABLE 3:** PDD parameters for both VHD and TB across all measured FS.

Unit	FS (cm^2^)	R50 (cm)	R80 (cm)	Ds (%)	D5 (%)	D10 (%)	D20 (%)
VHD	2 × 2	12.81	5.49	39.48	82.63	59.74	32.22
	3 × 3	13.24	5.79	39.35	84.07	61.24	33.13
	4 × 4	13.54	5.95	40.06	84.75	62.28	33.88
	5 × 5	13.95	6.12	45.93	85.45	63.47	34.99
	7 × 7	14.46	6.34	47.28	86.19	64.93	36.35
	10 × 10	15.04	6.54	49.57	86.74	66.27	37.91
	15 × 15	15.70	6.77	52.54	87.28	67.73	39.75
	20 × 20	16.07	6.86	55.18	87.42	68.39	40.72
	30 × 30	16.53	7.00	58.52	87.80	69.25	42.00
	40 × 40	16.69	7.06	59.89	87.91	69.56	42.42
TB	2 × 2	10.99	4.61	54.50	77.75	53.72	26.96
	3 × 3	11.35	4.80	59.27	78.90	55.06	27.54
	4 × 4	11.70	5.00	59.73	79.98	56.31	28.43
	5 × 5	12.03	5.16	60.59	80.87	57.37	29.23
	7 × 7	12.60	5.40	61.35	82.13	59.30	30.76
	10 × 10	13.24	5.66	63.25	83.14	61.38	32.67
	15 × 15	13.96	5.91	65.73	84.02	63.34	34.81
	20 × 20	14.44	6.07	67.79	84.68	64.53	36.18
	30 × 30	14.95	6.23	71.26	85.03	65.68	37.71
	40 × 40	15.24	6.30	72.96	85.36	66.12	38.48

Note: All measurements at SSD = 90 cm.

Abbreviations: Ds, surface dose was defined as the dose at 0.0 mm depth; Dx, percentage dose at x cm depth; Rx, depth at which x% dose is reached.

For surface dose, the Shapiro–Wilk test indicated a significant departure from normality (*W* = 0.757, *p* = 0.004); therefore, a Wilcoxon signed‐rank test was applied. TB delivered significantly higher surface doses than VHD across all FSs (mean Ds: TB = 63.64 ± 5.77% vs. VHD = 48.78 ± 7.72%; Wilcoxon W = 0, z = −2.803, *p* = 0.002, matched rank biserial *r* = −1.000; Hodges–Lehmann estimate = −14.07%). The effect size of |*r*| = 1.000 indicates that TB surface dose exceeded VHD in all ten paired FSs without exception, representing a consistent inter‐platform difference of 12.6–19.9 percentage points. For D10, the Shapiro–Wilk test confirmed normality (W = 0.857, *p* = 0.071). VHD showed significantly higher percentage depth dose at 10 cm depth than TB (mean VHD D10 = 65.29 ± 3.50% vs. mean TB D10 = 60.28 ± 4.56%; mean difference = 5.005 ± 0.352%; *t*(9) = 14.22, *p* < 0.001, Cohen's *d* = 4.498). A complete summary of all inferential statistics is provided in Table 4.

**TABLE 4:** Summary of inferential statistical analyses for inter‐platform PDD parameter and UCI comparisons (n = 10 FS for PDD parameters; n = 8 for UCI analyses).

Parameter	Mean VHD	Mean TB	Test	Statistic	p	Effect size	Direction
**R50 (cm)**	14.80 ± 1.41	13.05 ± 1.54	*Paired t‐test*	t(9) = 36.34	<0.001	*d* = 11.49	VHD > TB
**Ds (%)**	48.78 ± 7.72	63.64 ± 5.77	*Wilcoxon signed‐rank*	W = 0, z = −2.803	0.002	*r* = −1.000	TB > VHD
**D10 (%)**	65.29 ± 3.50	60.28 ± 4.56	*Paired t‐test*	t(9) = 14.22	<0.001	*d* = 4.498	VHD > TB
**UCI vs FS**	—	—	*Pearson r*	*r* = 0.916, n = 8	0.001	*r* ^2^ = 0.840	Positive
**UCI vs Depth**	—	—	*Repeated measures ANOVA*	F(1.911,13.38) = 1.348	0.292	η^2^ = 0.161	NS

*Note*: Values are mean ± SD. Normality was assessed by the Shapiro–Wilk test before parametric testing.

Effect size: Cohen's d for parametric tests; matched rank biserial *r* for Wilcoxon. For Ds, Hodges–Lehmann estimate = −14.07%.

Abbreviations: FS, field size; NS, not significant; UCI, Unflatness Conversion Index.

### 3.2 Beam profiles

**FIGURE 3:**
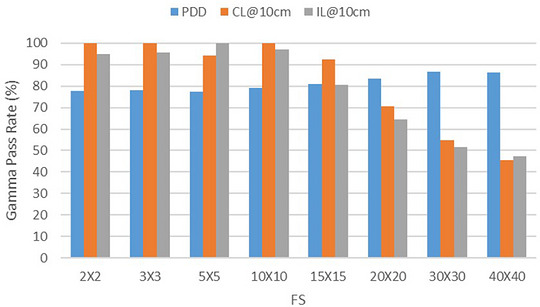
The Gamma index for PDD, CL, and IL profiles at a depth of 10 cm.

Gamma analysis at 10 cm depth showed high inter‐platform agreement at small FS, with CL profiles achieving 100% pass rates at 2 × 2, 3 × 3, and 10 × 10 cm^2^, and 94% at 5 × 5 cm^2^. Beyond 10 × 10 cm^2^, CL pass rates declined progressively to 92.5%, 70.5%, 55.0%, and 45.5% at 15 × 15, 20 × 20, 30 × 30, and 40 × 40 cm^2^, respectively. IL profiles peaked at 99% at 5 × 5 cm^2^ and remained above 95% up to 10 × 10 cm^2^, then declined to 80.5%, 64.5%, 52.0%, and 47.0% at larger FS. PDD pass rates were stable across the full field‐size range, between 78% at small fields and 86% at 30 × 30 and 40 × 40 cm^2^, with a gradual monotonic improvement as field size increased (Figure 3).

Unflatness values across all FSs and depths are presented in Table 5. VHD consistently exhibited higher unflatness values than TB, with the divergence increasing as FS grew. At 40 × 40 cm^2^, the unflatness range was 1.80–1.84 for VHD versus 1.63–1.68 for TB.

**TABLE 5:** Unflatness and UCI parameters for VHD and TB across FS and measurement depths.

FS (cm^2^)	Depth (cm)	VHD CL	VHD IL	TB CL	TB IL	UCI Mean	VHD vs TB (%)
2 × 2	5	1.10	1.10	1.06	1.06	**1.02**	1.89%
	10	1.10	1.10	1.06	1.06	**1.02**	1.89%
	20	1.11	1.11	1.05	1.05	**1.03**	3.33%
3 × 3	5	1.07	1.07	1.03	1.03	**1.01**	1.00%
	10	1.07	1.07	1.03	1.03	**1.02**	2.43%
	20	1.07	1.07	1.03	1.03	**1.02**	2.43%
5 × 5	5	1.04	1.04	1.04	1.04	**1.00**	−0.48%
	10	1.04	1.04	1.04	1.04	**1.00**	−0.48%
	20	1.05	1.05	1.03	1.03	**1.01**	1.46%
10 × 10	5	1.07	1.07	1.06	1.06	**1.01**	0.94%
	10	1.08	1.08	1.13	1.13	0.96	−4.42%
	20	1.17	1.17	1.14	1.14	**1.02**	2.19%
15 × 15	5	1.23	1.23	1.12	1.12	1.09	9.38%
	10	1.24	1.24	1.20	1.20	**1.03**	3.33%
	20	1.26	1.26	1.22	1.22	**1.03**	2.87%
20 × 20	5	1.33	1.33	1.26	1.26	**1.05**	5.16%
	10	1.35	1.35	1.28	1.28	**1.05**	5.08%
	20	1.37	1.37	1.30	1.30	**1.05**	5.00%
30 × 30	5	1.56	1.56	1.43	1.43	1.09	8.74%
	10	1.57	1.57	1.45	1.45	1.08	8.28%
	20	1.60	1.60	1.48	1.48	1.08	7.77%
40 × 40	5	1.80	1.80	1.63	1.63	1.10	10.43%
	10	1.82	1.82	1.65	1.65	1.10	10.00%
	20	1.84	1.84	1.68	1.68	1.09	9.23%

*Note*: Colour coding: Green (UCI ≈ 1.00) = near‐equivalent unflatness; Amber (UCI 1.04–1.07) = moderate divergence; Red (UCI ≥ 1.08) = substantial divergence; Blue = TB marginally more unflat. Pearson *r* = 0.916 (*p* = 0.001) between UCI_Mean and FS; depth effect not significant (repeated measures ANOVA *p* = 0.292).

Pearson correlation analysis confirmed a strong positive relationship between UCI_Mean and FS (*r* = 0.916, n = 8, *p* = 0.001, 95% CI [0.595, 0.985]; *r* ^2^ = 0.840), indicating that FS accounts for 84.0% of the variance in UCI and that beam unflatness divergence between the two platforms increases monotonically with aperture size. UCI values were close to unity at small fields (UCI_Mean ≈ 1.015 at 2 × 2 cm^2^), indicating near‐equivalent unflatness, then increased progressively to 1.050–1.104 at large FSs (20 × 20–40×40 cm^2^), confirming that VHD was consistently 5%–10% more unflat than TB across the large‐field clinical range.

Repeated measures ANOVA revealed no statistically significant effect of measurement depth (5, 10, and 20 cm) on UCI values (*F*(1.911, 13.38) = 1.348, *p* = 0.292, η^2^ = 0.161), with mean UCI values of 1.046 ± 0.042, 1.033 ± 0.044, and 1.041 ± 0.029 at 5, 10, and 20 cm depth, respectively. Mauchly's test confirmed the sphericity assumption was met (*W* = 0.954, *p* = 0.867), and the Greenhouse‐Geisser correction was applied (ε = 0.956). These findings confirm that UCI is a depth‐independent metric, implying that a single reference depth measurement is sufficient for cross‐platform unflatness comparisons.

Slope parameters are presented in Table 6. For both linacs, slope values decreased progressively as FS increased from 2 to 40 cm^2^, consistent with improving dose uniformity at larger apertures. At equivalent FSs and depths, TB consistently showed lower slope values than VHD, indicating smoother dose gradients across the beam profile. In VHD, CL slopes substantially exceeded IL slopes for small fields (e.g., 3.25 vs. 1.80 at 2 × 2 cm^2^, 5 cm depth), whereas in TB the CL–IL difference was markedly less pronounced.

**TABLE 6:** Slope parameters for VHD and TB across FS and measurement depths.

Unit	FS (cm^2^)	CL 5 cm (%)	CL 10 cm (%)	CL 20 cm (%)	IL 5 cm (%)	IL 10 cm (%)	IL 20 cm (%)
VHD	2	3.25	2.97	2.79	1.80	1.67	1.54
	3	1.25	1.14	1.10	0.82	0.79	0.70
	5	0.51	0.49	0.46	0.44	0.43	0.39
	10	0.40	0.41	0.39	0.38	0.40	0.38
	15	0.47	0.46	0.43	0.46	0.46	0.42
	20	0.51	0.50	0.46	0.51	0.50	0.47
	30	0.56	0.53	0.49	0.56	0.54	0.50
	40	0.56	0.52	0.59	0.60	0.57	0.53
TB	2	2.20	2.13	1.88	2.20	2.15	2.05
	3	0.71	0.67	0.61	0.86	0.83	0.77
	5	0.40	0.40	0.34	0.41	0.43	0.35
	10	0.32	0.35	0.33	0.34	0.36	0.33
	15	0.37	0.37	0.35	0.38	0.37	0.36
	20	0.40	0.39	0.38	0.40	0.38	0.38
	30	0.45	0.43	0.41	0.40	0.43	0.40
	40	0.48	0.45	0.42	0.48	0.46	0.43

**FIGURE 4:**
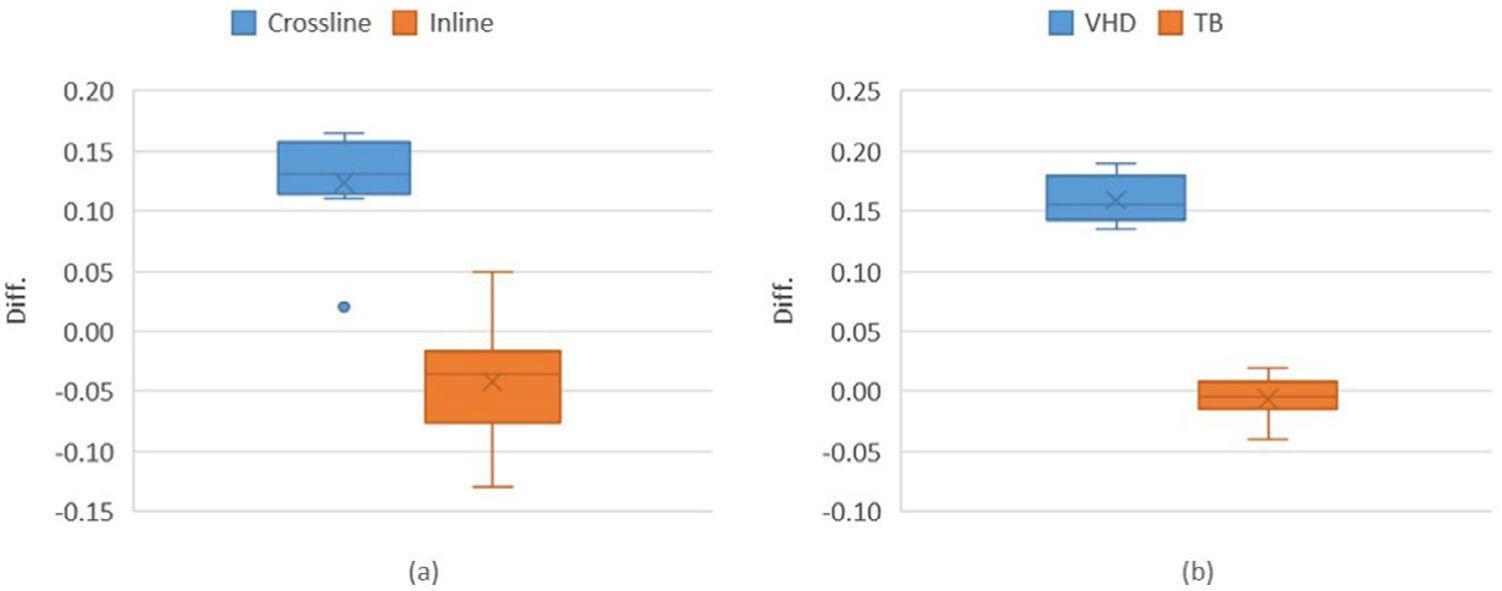
Penumbra differences at 5 cm depth. (a) Inter‐platform differences (VHD − TB) in CL and IL directions. (b) Intra‐machine CL‐to‐IL asymmetry for VHD and TB.

At 5 cm depth, CL penumbra differences were consistently positive across all FS (median Diff. = +1.33 mm; IQR: +1.10 to +1.58 mm), while IL differences were predominantly negative (median Diff. = −0.45 mm; IQR: −0.75 to −0.20 mm), indicating that VHD exhibited wider CL but narrower IL penumbra than TB (Figure 4a). A single CL outlier at +0.22 mm was recorded at 5 × 5 cm^2^. Intra‐machine analysis revealed that VHD carried a large internal CL−IL asymmetry (median Diff. = +1.65 mm; IQR: +1.50 to +1.85 mm), whereas TB demonstrated near‐zero asymmetry between planes (median = −0.02 mm; IQR: −0.30 to +0.20 mm) (Figure 4b).

### 3.3 Output factors (OFs)

Measured OFs for both platforms across the full FS range are presented in Table 7. Both linacs demonstrated a monotonic increase in OF with increasing FS, normalized to unity at the 10 × 10 cm^2^ reference field. For small FSs (1 × 1 to 5 × 5 cm^2^), TB yielded higher OFs than VHD, with the largest absolute discrepancy of 0.064 recorded at 1 × 1 cm^2^ (TB = 0.741 vs. VHD = 0.677). The relationship reversed at 7 × 7 cm^2^, where VHD produced a marginally higher OF (0.964 vs. 0.951). For large FSs (15 × 15 to 40 × 40 cm^2^), TB again produced higher OFs, with a maximum absolute difference of 0.042 at 40 × 40 cm^2^ (TB = 1.133 vs. VHD = 1.091).

**TABLE 7:** OFs for TB and VHD across FS from 1 × 1 to 40 × 40 cm^2^. Both platforms were normalized to unity at 10 × 10 cm^2^.

Field size (cm × cm)	TB OF	VHD OF
1 × 1	0.741	0.677
2 × 2	0.859	0.835
3 × 3	0.893	0.884
4 × 4	0.918	0.915
5 × 5	0.936	0.937
7 × 7	0.951	0.964
10 × 10	1.000	1.000
15 × 15	1.051	1.039
20 × 20	1.083	1.061
30 × 30	1.119	1.084
40 × 40	1.133	1.091

*Note*: Small‐field measurements (≤5 × 5 cm^2^) performed using IBA RAZOR chamber with IAEA TRS‐483 correction factors (*k* = 1.004 at 1 × 1 cm^2^ to *k* = 1.000 at 5 × 5 cm^2^). Standard‐field measurements (≥7 × 7 cm^2^) performed using IBA CC13 chamber.

### 3.4 Treatment planning study

Plan parameters for both platforms across the fours target sizes are summarized in Table 8. As plans were normalized to D95% for both platforms. Monitor units (MU) required to deliver the prescribed dose were higher for TB than VHD at both target sizes (small: 3002 vs 2553 MU; large: 1830 vs 1643 MU), with the relative difference being 17.6% for the small target and 11.4% for the large target. The surface dose was higher for TB at both the small (46.82% vs. 37.23%) and large (43.25% vs. 32.98%) targets. The homogeneity index was 0.383 for VHD versus 0.416 for TB at the small target, and 0.207 for VHD versus 0.188 for TB at the large target. D2% was 13.703 Gy for VHD versus 14.380 Gy for TB at the small target and 11.314 Gy for VHD versus 11.122 Gy for TB at the large target. Conformity indices were 0.896 (VHD) and 0.915 (TB) for the small target, and 0.872 (VHD) and 0.868 (TB) for the large target.

**TABLE 8:** Plan parameters for VHD and TB across the four target sizes.

	1 cm			3 cm			6 cm			12 cm		
Target Metric	VHD	TB	Diff. %	VHD	TB	Diff. %	VHD	TB	Diff. %	VHD	TB	Diff. %
D2% (Gy)	13.7	14.4	−4.9	13.9	13.5	2.6	11.3	11.1	1.7	11.8	11.6	1.8
GI	5.26	4.21	19.9	3.65	3.86	−5.5	3.87	4.02	−3.9	3.10	3.19	−2.7
HI	0.38	0.42	−8.5	0.39	0.37	5.0	0.21	0.19	8.8	0.24	0.22	8.5
CI	2.05	2.22	−8.1	1.48	1.47	0.7	0.96	0.95	1.2	0.95	0.94	0.5
Surface dose (%)	3.7	4.7	−25.8	3.7	4.5	−21.5	3.3	4.3	−31.1	7.9	9.1	−15.9
MU	2555	3003	−17.6	2201	2437	−10.7	1644	1831	−11.4	1596	1691	−5.9

## 4 DISCUSSION

Accurate characterization of PDD, beam profiles, and OFs is essential for reliable TPS beam modelling and clinical application. In this study, commissioning parameters of 6 MV FFF beams from the VHD and TB platforms were compared under equipment‐equivalent conditions, enabling direct attribution of observed differences to platform‐specific beam characteristics rather than measurement‐related variability.

The PDD analysis demonstrated systematic inter‐platform differences, with VHD exhibiting greater beam penetration than TB (ΔR50 ≈ 1.75 cm), consistent with previously reported differences in beam generation between the two platforms.^8^, ^10^, ^24^ This behavior reflects a stable difference in effective beam quality and is directly relevant for TPS modelling, where depth‐dose characteristics determine energy spectrum representation and influence calculated dose distributions at depth. The higher surface dose observed for TB is similarly consistent with prior reports and reflects differences in build‐up region behavior, which are clinically relevant in high‐dose‐per‐fraction treatments.^8^

The treatment planning study provides insight into how these commissioning differences propagate into calculated dose distributions. The deeper penetration of VHD translated into reduced **MU** requirements for equivalent target coverage, consistent with increased dose deposition efficiency at depth. Conversely, the higher surface dose associated with TB was preserved in the calculated plans across all target sizes, indicating that differences in the build‐up region are maintained within the TPS beam model. These observations demonstrate that commissioning‐level differences are not only measurable but are systematically reflected in clinically relevant plan parameters.

A target‐size‐dependent behavior was observed at the plan level, with VHD demonstrating more favorable homogeneity for the smallest target and TB performing slightly better for larger targets. This finding is consistent with the field‐size dependence of beam profile characteristics described in previous studies.^9^, ^10^, ^24^ In this context, the UCI provides a compact descriptor of inter‐platform divergence, showing a strong correlation with field size and confirming that profile differences increase with aperture while remaining independent of measurement depth. The agreement between UCI trends and plan‐level homogeneity suggests that off‐axis beam characteristics contribute differently across field‐size regimes and influence dose uniformity in a nonlinear manner.

These results indicate that commissioning parameters such as depth‐dose characteristics and beam profiles interact within the TPS beam model to determine clinically relevant outcomes, including delivery efficiency, surface dose, and dose homogeneity. In particular, the transition from small to larger fields appears to be a region in which differences in beam architecture become increasingly influential.

From a clinical perspective, the present study is not intended as a generalized vendor comparison but as a practical framework for addressing a common scenario in radiotherapy departments operating multiple linear accelerators. The aim was to quantify dosimetric differences between two clinically used machines and to assess their potential impact on planning and treatment delivery. This approach is directly relevant for optimizing patient allocation and treatment strategy across available platforms within the same institution.

The profile analysis supports this interpretation. VHD exhibited higher unflatness and a systematic crossline‐to‐inline asymmetry consistent with the Agility™ MLC design, while TB showed more symmetric profiles and lower slope values, indicating smoother gradients. These findings are in agreement with previously reported observations.^10^, ^11^ Output factor differences followed expected trends, with the largest discrepancies observed in the small‐field region, consistent with known sensitivity to detector response and head‐scatter conditions and with published multicenter studies.^23^, ^25^, ^26^, ^27^ At larger field sizes, differences remained within internationally accepted commissioning variability ranges.^23^, ^26^

Several limitations should be considered. Each platform was represented by a single unit installed at a different institution; although identical instrumentation reduced measurement‐related variability, unit‐specific factors cannot be fully excluded. Statistical comparisons across field sizes should also be interpreted with caution, as measurements at different apertures are not fully independent; in this study, statistical metrics are used primarily to describe consistency and trends across the clinical range. In addition, the treatment planning study was limited to a forward‐planned 3D‐CRT configuration. While this approach isolates beam model effects, further investigation using inverse‐planned techniques is warranted to evaluate the impact on optimization and clinical endpoints.

Overall, this study demonstrates that platform‐dependent differences in FFF beam characteristics are measurable under standardized conditions and are reflected in plan‐level parameters. The combined use of a harmonized measurement methodology, a simple descriptor such as UCI, and targeted planning study provides a practical framework for assessing and managing inter‐machine variability in routine clinical practice.

## 5 CONCLUSION

A harmonized measurement approach demonstrated systematic differences between VHD and TB FFF beams in penetration, surface dose, and field‐size‐dependent profile characteristics. These differences were shown to propagate into treatment planning across four target sizes (1–12 cm), influencing **MUs**, surface dose, dose gradient, and dose homogeneity in a target‐size‐dependent manner, with VHD demonstrating advantages for small targets and TB for larger ones. The UCI, depth‐independent and strongly correlated with field size (*r* = 0.916), provides a simple cross‐platform unflatness comparison tool.

The study provides a practical framework for quantifying dosimetric differences between linear accelerators within the same department, supporting machine‐specific planning strategies and patient allocation in multi‐platform clinical settings.

## AUTHOR CONTRIBUTIONS

**Mustafa K. Al‐Aseebee**: Conceptualization, data collection, data analysis, methodology, writing the original paper draft. **Hussien Mraity**: Supervision, writing, review, and editing. **Marco Esposito**: Methodology, supervision, writing, review, and editing.

## FUNDING INFORMATION

The authors have nothing to report.

## CONFLICT OF INTEREST STATEMENT

The authors declare no conflicts of interest
